# A case of adrenocortical oncocytic carcinoma arising in ectopic adrenal tissue: a multidisciplinary diagnostic challenge

**DOI:** 10.3332/ecancer.2020.1135

**Published:** 2020-11-05

**Authors:** Nikita Wadhwani, Daniel Mais, Dharam Kaushik, Mio Kitano

**Affiliations:** 1 University of Alabama at Birmingham, Birmingham, Alabama 35233, USA; 2 Associate Professor, Department of Pathology, University of Texas Health San Antonio, San Antonio, Texas 78229, USA; 3 Assistant Professor, Department of Urology, University of Texas Health San Antonio, San Antonio, Texas 78229, USA; 4 Assistant Professor, Division of Surgical Oncology & Endocrine Surgery, University of Texas Health San Antonio, San Antonio, Texas 78229, USA; a https://orcid.org/0000-0003-4302-5921

**Keywords:** adrenal, endocrine surgery, ectopic adrenal

## Abstract

Adrenocortical oncocytic neoplasm arising in ectopic adrenal tissue is a rare finding and presents as a unique diagnostic challenge. We report a case of a 26-year-old female who presented with vague left-sided abdominal pain and a large left retroperitoneal mass. She underwent exploratory laparotomy and resection of the mass and was diagnosed with extra-adrenal adrenocortical oncocytic carcinoma.

## Background

Adrenocortical carcinoma is a rare, typically aggressive cancer with an incidence of 0.7 per 2 million cases annually [1]. Adrenocortical oncocytic neoplasm (AON) is a rare and diagnostically challenging subtype of adrenocortical tumours [2]. Most AONs are nonfunctional and, therefore, are discovered incidentally or present with vague abdominal or back pain. AONs are diagnosed predominantly in females with an average age of 44 years at diagnosis [3]. We report a case of a 26-year-old female who presented with vague left-sided abdominal pain and a large left retroperitoneal mass on CT, which was eventually diagnosed as extra-adrenal adrenocortical oncocytic carcinoma (AOC).

## Case presentation

A 26-year-old Hispanic female presented to the Emergency Department (ED) with a 2-day history of abdominal pain. She described her pain as dull, progressive and localised to the left abdomen. She denied any changes to her usual state of health, especially with respect to her weight, appetite, nausea, vomiting or bowel habits. Her past medical history was insignificant. Last menstrual period was 2 weeks prior to presentation. Her vital signs and routine blood workup, including complete blood count and comprehensive metabolic panel, were all within normal limits.

**Figure 1.**
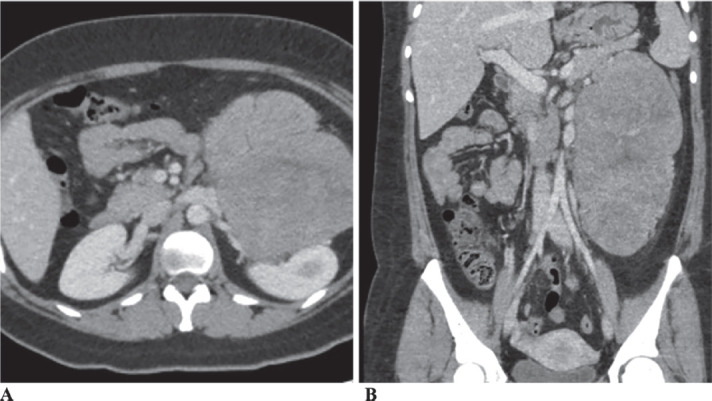
CT Abdomen (1A-Axial section and 1B-Coronal section) showing a 21 × 15 cm mass abutting the left kidney, left renal vessels, distal pancreas and spleen.

She underwent a computerised tomography (CT) scan, which revealed a large 21 × 15 cm mass, which appeared to be abutting the left kidney, left renal vessels, distal pancreas and spleen (Figure 1). She was admitted for further workup. Tumour markers including CEA, CA19-9, AFP and CA-125 were all within normal limits. Hormonal workup, including plasma free metanephrines, 24-hour cortisol, renin, aldosterone levels, was unremarkable. She underwent a core needle biopsy of the mass. Despite review by pathologists across multiple institutions, the results remained inconclusive. A preliminary diagnosis of a non-functioning paragangliomas (PGL) was made after an electron microscopic review. Patient was empirically started on Doxazosin, a selective alpha-blocker, 2 mg at bedtime for 4 weeks preoperatively for presumed PGL. The patient was subsequently taken to the operating room for exploratory laparotomy. After careful dissection, the mass was seen lying anterior to the left kidney. There was a clear demarcation between the structures; there was no evidence of invasion into the left kidney or its vasculature, and the left adrenal gland was uninvolved. The mass was carefully dissected, preserving the left kidney, ureter and renal vasculature. Postoperative period was uneventful, and she was discharged on postoperative day 4.

**Figure 2.**
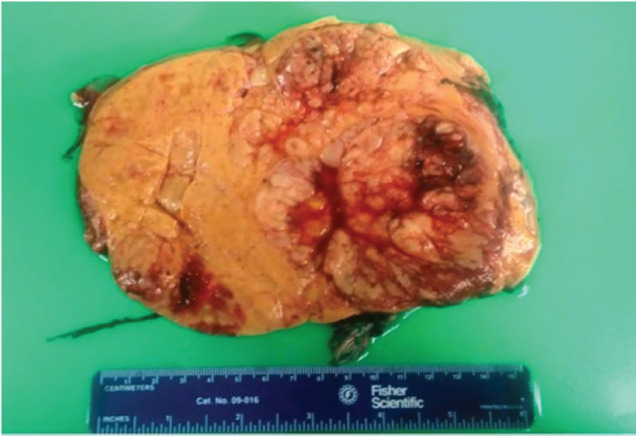
Gross specimen of the mass measuring 28 × 18 × 10.5 cm and weighing 2,250 g.

**Figure 3.**
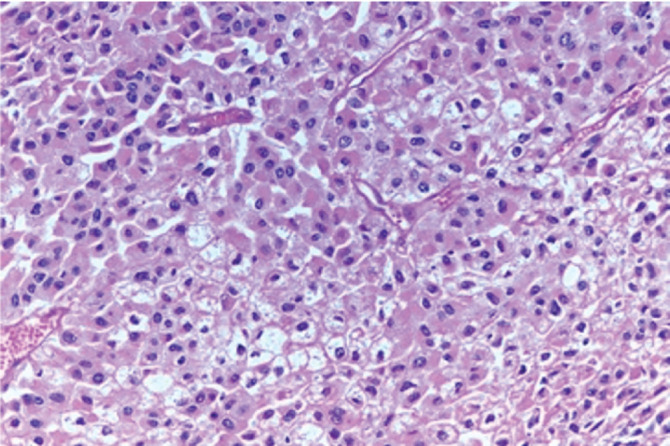
Histopathology of the mass showing a diffuse sheet-like growth pattern with a compact eosinophilic cytoplasm throughout, resembling oncocytic cells.

Grossly, the mass measured 28 × 18 × 10.5 cm and weighed 2,250 grams (Figure 2). Histopathology revealed a diffuse sheet-like growth pattern with a compact eosinophilic cytoplasm throughout, resembling oncocytic cells (Figure 3). Immunohistochemistry showed multifocal positivity for pan-cytokeratin, SF1 and melan-A while SMA, desmin, HMB45, inhibin and calretinin were negative. The possibility of PGL was ruled out based on histopathological analysis of the specimen. After a consensus review by several pathologists at various institutions, the mass was eventually diagnosed as AOC on the basis of the microscopic appearance of oncocytic cells, atypical mitotic figures and high Ki-67 expression. Ki-67 expression was approximately 10%–15%, which is malignant per Helsinki system. A small focus of residual normal adrenal tissue was seen on the pathology supporting the diagnosis. As both adrenal glands were uninvolved, this carcinoma is believed to have originated from ectopic adrenal tissue.

Adjuvant chemotherapy was recommended based on the pathological diagnosis; however, the patient declined to receive any further treatment. She is under surveillance and has had no evidence of recurrence at 30 months follow-up.

## Discussion

AONs are an extremely rare subtype of adrenocortical carcinoma (ACC) that were first described in 1986 [4]. A total of 162 cases of AON have been reported in the literature till date [4]. Most of these tumours are discovered incidentally and are usually benign [5]. AONs do not have a specific age predilection, and the age at diagnosis ranges from 27 to 72 years [4]. They occur more frequently in females (F/M: 2.5/1) and on the left side [4]. AONs are classified as oncocytoma (AO), oncocytic neoplasm of uncertain malignant potential (AONUMP) and oncocytic carcinoma (AOC) [4]. Only 32 cases of AOC, including the current case, have been reported to date [4].

Ectopic adrenal tissue is relatively common existing in approximately 6% of the population [6, 7]. Since the adrenal gland is derived from primordial mesenchyme adjacent to the dorsal mesentery and urogenital structures, most of the ectopic adrenal tissue is found along this path of embryonic migration [6]. Cases of ACC have been known to occur in ectopic adrenal rests, but the presence of AOC in an ectopic adrenal tissue is rare.

The clinical presentation of AOCs is usually due to mass effect on surrounding structures as only 17% of the oncocytic neoplasms are hormonally active [5]. Therefore, they can reach large sizes prior to diagnosis. Furthermore, AOC arising outside of conventional locations imposes additional diagnostic challenges, like the current case.

Unlike most adrenocortical malignancies, the size of the mass and the imaging findings on CT or MRI are not pathognomonic for AOC. Although the percentage washout on CT has been correlated to the differentiation of benign versus malignant AON [3], this finding does not have proven statistical significance and requires a high index of preoperative suspicion to draw a conclusion of AON amongst other adrenal tumours on CT. Fine needle aspiration and core biopsies are seldom helpful to define AON preoperatively [3, 4].

The workup of these oncocytic tumours is similar to adrenal incidentaloma. The final diagnosis is made after a histopathological assessment of the tumour. On gross examination, AOCs are large, round, encapsulated, brown-yellow and well-circumscribed masses with an average diameter of 8 cm and a thin rim of the normal adrenal gland [4]. Microscopically, AOCs are characterised by diffuse proliferation of oncocytic cells, i.e., polygonal cells with abundant granular and eosinophilic cytoplasm, capsular and/or vascular invasion, necrosis and several mitotic figures [4].

However, it should be emphasised that the determination of the oncocytic character of the tumour cells represents another diagnostic hurdle. One of the reasons for this is that the determination of oncocytic character is based on subjective morphological criteria [2]. Our case was no exception, thereby requiring consultation with various pathologists. Such diagnostic subjectivity is a major limitation to the use of a histoprognostic score [2]. Currently, AONs are classified based on the modified Weiss criteria, also known as the Lin–Weiss–Bisceglia (LWB) system, to elucidate their malignant potential [8], as this score does not include relatively more subjective histologic criteria, as in the original Weiss score [2]. According to the LWB system, AOC is characterised by the presence of one of the following three major criteria: mitotic rate >5/50 high-power field (HPF), atypical mitotic figures and/or venous invasion [8]. Recently, Helsinki score has been validated for ACC variants, in particular, the oncocytic variant [9]. This score includes the Ki-67 proliferation index, which has an independent prognostic value for AONs. Ki-67 is correlated with the mitotic index and is usually ≥5% for tumours graded malignant and ≤ 4% for borderline or benign tumours [8].

Regardless of the scoring, patients with AOC have a better prognosis when compared to the conventional adrenocortical carcinomas. Adrenocortical carcinomas are aggressive, with an estimated median survival of 8–32 months. AOC, on the other hand, has a comparatively favourable median survival of 58 months [1, 2].

AOC does not have well-established protocols regarding management given the limited data available on patient outcomes. Frequently, treatment follows the same course as in conventional adrenocortical carcinoma [4, 5, 10]. An open or laparoscopic approach can be employed for ipsilateral adrenalectomy with or without concomitant nephrectomy, depending on the extent of the tumour. The goal is to obtain complete surgical resection (R0) often followed by adjuvant chemotherapy and tumour bed radiation, depending on the final pathological findings.

AONs have a reported recurrence rate of 16%, with most recurrences occurring within first 5 years [3]. There is no specific surveillance schedule for AONs, and most patients are followed according to a standard protocol for ACC. In addition, due to the rarity of AOC, there is currently no standardised neoadjuvant or adjuvant therapy. The most common chemotherapeutic agent used in both ACC and AOC is Mitotane, a derivative of the insecticide dichlorodiphenyltrichloroethane, with adrenolytic and cytotoxic activity [9]. Mitotane metabolites inhibit several enzymes in the adrenocortical steroidogenesis pathway, mainly CYP11A1 and CYP11B1. Its use in the treatment of AOC remains controversial given the different rates of tumour recurrence and hormonal activity compared to ACC [5, 11]. In our case, the patient declined to receive chemotherapy. She is under surveillance, and she has no evidence of recurrence at 30 months.

## Conclusion

AOC are a rare clinical entity, with only 32 cases reported to date. The present case emphasises the multidisciplinary diagnostic challenges faced during detection of AOC as they do not have any radiological or clinical pathognomonic features, in addition to the ectopic location of the tumour in this case. Thus, oncocytic variants should be in the differential when dealing with tumours of unknown origin along the path of embryonic migration of the adrenals. LWB scoring or the recently validated Helsinki score is used to elucidate the malignant potential of ACC and AOC. Management is aimed at achieving an R0 surgical resection, followed by adjuvant chemotherapy and/or radiation.

## Conflicts of interest

None.

## Financial support/source of funding

None.

## Authors’ contributions

The preparation, design and editing of the original draft were done by N.W. D.M. and D.K. reviewed and edited the manuscript. M.K. planned and supervised the project, editing and critical revision. All authors have read and approved the final version of the manuscript.
